# No Own-Age Advantage in Children’s Recognition of Emotion on Prototypical Faces of Different Ages

**DOI:** 10.1371/journal.pone.0125256

**Published:** 2015-05-15

**Authors:** Sarah Griffiths, Ian S. Penton-Voak, Chris Jarrold, Marcus R. Munafò

**Affiliations:** 1 School of Experimental Psychology, University of Bristol, Bristol, United Kingdom; 2 Medical Research Council Integrative Epidemiology Unit (MRC IEU) at the University of Bristol, Bristol, United Kingdom; 3 UK Centre for Tobacco and Alcohol Studies, University of Bristol, Bristol, United Kingdom; University of Dundee, UNITED KINGDOM

## Abstract

We test whether there is an own-age advantage in emotion recognition using prototypical younger child, older child and adult faces displaying emotional expressions. Prototypes were created by averaging photographs of individuals from 6 different age and sex categories (male 5–8 years, male 9–12 years, female 5–8 years, female 9–12 years, adult male and adult female), each posing 6 basic emotional expressions. In the study 5–8 year old children (n = 33), 9–13 year old children (n = 70) and adults (n = 92) labelled these expression prototypes in a 6-alternative forced-choice task. There was no evidence that children or adults recognised expressions better on faces from their own age group. Instead, child facial expression prototypes were recognised as accurately as adult expression prototypes by all age groups. This suggests there is no substantial own-age advantage in children’s emotion recognition.

## Introduction

The ability to recognise emotions in other people from their facial expressions is an important skill for social development [1]. It has been found that children do not reach adult levels of performance on emotion recognition tasks until late adolescence [1, 2]. However, when comparing different age groups on emotion recognition performance, most studies have used adult faces, failing to take in to account the effect this may have on the performance of children, who obviously do not belong to this age category.

It has been established that people are better at processing the faces of individuals belonging to their in-group, compared to the faces of individuals belonging to an out-group. In studies of face recognition, participants are more accurate at recognising faces of individuals belonging to their own cultural group [3] and at recognising faces of individuals belonging to their own age-group [4, 5]. There is also some evidence that people find it easier to recognise emotional expressions on faces of individuals belonging to their in-group. In a meta-analysis Elfenbein and Ambady (6) concluded that expression recognition is more accurate when the perceiver and the expresser are members of the same cultural group.

There are two predominant theories for why in-group advantages in face processing exist, which may be applied to recognition of facial emotions, as well as recognition of facial identity. One theory is that people show increased attention to the faces of individuals from their in-group and this increased attention improves processing [7]. An alternative theory is that greater experience with in-group faces leads to perceptual expertise for coding certain types of facial features [3]. There is evidence that there are subtle differences in how expressions are presented on different age faces due to age-related changes in facial characteristics [8]. Therefore lack of experience with a certain age group may feasibly decrease the ability to accurately recognise the emotions of members of that group.

To date, the few studies that have looked for own-age biases in emotion recognition have compared younger and older adults [9, 10]. Two of these studies found no support for an own-age bias in emotion recognition, instead finding that both younger adults and older adults find it easier to recognise expressions on younger adult faces [9, 11]. One larger study did find some evidence for an own-age bias, with younger adults performing less well than older adults at recognising certain expressions on older adults’ faces. The authors suggest that the failure to find this effect in previous studies may have been due to lack of power as a result of small sample sizes [10].

To our knowledge, no previous studies have looked specifically at whether there is an own-age bias in children’s emotion recognition. To fill this gap we aimed to look for evidence of an own-age bias in children’s emotion recognition using stimuli with prototypical children’s facial expressions. Prototypes are created using computer graphics techniques to average shape and colour information from photos of a number of individuals who share certain characteristics (e.g., age, ethnicity, attractiveness or emotional expression) to isolate facial characteristics of interest [12]. For the current study we created faces typical of a certain age group, displaying 6 different facial expressions. Creating prototypes instead of using photos of individuals’ faces increases stimulus control by removing unwanted variation in other facial characteristics that may affect performance. This is particularly advantageous in studies where it is not possible to show a large number of faces due to limitations in participants’ attention, such as with children, since the fewer images there are, the larger the effect of extraneous variables in individual images is likely to be.

Emotion recognition is still developing throughout childhood and into adolescence [1, 2, 13, 14]. We therefore expected an increase in emotion recognition performance with age. However, while there is a general consensus that the ability to recognise happiness reaches adult levels in early childhood [1, 2], the age at which the recognition of other emotions reaches adult levels is disputed. For example, some studies have reported no improvement for anger recognition between the ages of 4 and 15 years, suggesting recognition of this emotion may mature early [14, 15], whereas others report improvements in anger recognition into adolescence [2, 16]. Similarly, some studies show no improvement in disgust recognition between 4 and 18 years [16], whereas others suggest improvement in disgust recognition into adolescence [14]. Therefore in addition to our focus on an own-age bias, we were also interested in looking at patterns of developmental improvement in recognition of different emotions.

In summary, in the current study we developed a set of prototypical child faces of each sex and two age groups (4–8 and 9–13 years), displaying 6 emotional expressions (happy, sad, angry, fearful, surprised and disgusted). We then tested whether there is an own-age advantage by comparing the accuracy of younger children, older children and adults at labelling expressions on our younger child and old child face prototypes as well as an existing set of adult face prototypes displaying the same 6 emotional expressions.

## Methods

### Participants

All participants were recruited from visitors to At-Bristol, a science museum for children in Bristol, United Kingdom. Participants were recruited in person by researchers during their visit to the museum. Written consent was obtained from adults for their own participation, and from parents for their children’s participation. This study was approved by the University of Bristol Faculty of Science Research Ethics Committee. Permission to carry out recruitment and testing in At-Bristol’s privately owned exhibition space was obtained from the Learning Team at At-Bristol.

A total of 211 child and adult visitors participated in the study. Data on 7 participants were lost due to technical error, 3 did not complete the task and 4 children were excluded due to parents helping during the task. Exact age data were missing for 5 participants, however it was noted that 3 of these were adults so these were included in the adult age group in the analysis, while the remaining 2 were not included in any group. This left a total of 195 participants for analysis. Demographic information can be found in Table 1.

**Table 1:** Participant demographics.

	Younger Children	Older Children	Adults
n	33	70	92
Male/female	17/16	25/45	30/61
Age range (years)	5–8	9–13	14–78
Age mean (years)	7	10	38
Age SD (years)	0.8	1.3	13.4

### Materials

#### Image collection

A total of 182 children (98 female, 54%) between the ages of 3 and 15 years (M = 8 years, SD = 2.57) were recruited from visitors to At-Bristol, 6 months prior to the main study. None of the participants in the main study reported having taken part in the photograph collection, although this could not be independently confirmed as the identities of the participants were not kept for ethical reasons. Participants were recruited in person during their visit to the museum. All parents signed a consent form allowing their children to take part in the photograph collection.

#### Procedure

The photography session took place in a booth created from black stage curtains on scaffolding (approximately 2 × 2 meters) in a corner of the exhibition space. Participants sat on a stool in the booth, in front of a light grey backing board. A Canon D450 digital SLR camera was used to take the photos with flash at a resolution of 3872 by 2592 pixels. Children were asked to imagine something that would make them feel each of the 6 emotions (happy, sad, angry, surprised, fearful and disgusted) in turn and then pull the face that they would pull if they were feeling that emotion. In cases where the children were unsure of what an emotion was, the experimenter gave examples of relevant scenarios (e.g., “Think about eating your least favourite food/having an argument with your brother and show me the face you would pull”). If they were wearing glasses or hats they were asked to remove these before taking part. The whole session lasted about 5–10 minutes.

#### Image selection

Eleven participants’ parents did not consent to the photographs of their children being stored. This left images of 171 children that could be used. Children who were not of European ethnicity were excluded as prototyping is more successful if faces all have similar colouration and facial features. In order to keep the age of faces in a narrow range, participants under 5 and over 12 years were also excluded. The remaining participants were split into 4 groups: girls aged 5–8 years (N = 53), boys aged 5–8 years (N = 43), girls aged 9–12 years (N = 28) and boys aged 9–12 years (N = 32). Images of the children in each group were rated by on one of the researchers (SG) for how suitable the photos were for making prototypes. This included checking the children were facing the camera, did not have anything obscuring their faces (e.g., long hair) and were making the correct expression. Ratings were out of 3, with 3 being unsuitable, 2 being suitable, and 1 being very suitable. Any participants with a suitability rating of 3 for any emotion were excluded. For the rest, a total suitability score across all emotions was calculated by adding up the 6 individual emotion scores for each participant. The images from the best scoring 15 participants in each of the four age/sex sets were then taken to be used to make prototypes.

#### Prototype creation

Prototype faces of the 4 age/sex groups pulling each of the 6 expressions were created using Psychomorph [12]. Each of the 360 images was delineated by placing 172 points in the same position on the features of each face. This allowed colour and texture information to be averaged across all 15 faces [12]. The intensity of the expressions was then increased. In order to do this, all 90 images from each age/sex group were averaged to produce 4 emotion ambiguous prototypical faces [17]. The linear vector difference of each delineation point on the emotion ambiguous prototype compared to emotion expression prototype was then increased to effectively ‘caricature’ the expression, making it more intense [18]. Colour and texture information from the emotional prototype was then rendered into this new shape. The level of caricature for each expression was chosen so that the face did not distort or look unnatural. An ambiguous rather than a neutral expression was used for caricaturing because the intention was to increase the perceptual difference between the 6 expressions. It makes sense therefore to choose an average of all expressions as the starting point, rather than a neutral expression which is often perceived as similar to an angry expression [19, 20].

#### Study stimuli

Original prototypes and caricatured prototypes for each of the 6 expressions on the 4 prototypical child faces described above were included in the study. Both the original prototypes and caricatured prototypes were included to check that increasing the intensity of the expression had been successful in making the expressions more recognisable.

In addition to the child face prototypes, existing adult face prototypes were included. These stimuli had been developed and used in other unpublished studies. They were created from photos of individuals between the ages of 19–23 who were recruited from the undergraduate population at the University of Bristol through notices on campus. Individuals took part in a 20 minute photograph session and were reimbursed £5. Information on the exact age of these individuals is not available but all were within the narrow age range noted above. Table 2 summarises information about the faces used to construct the adult prototypes and those used to construct the child prototypes.

**Table 2:** Faces used for prototypes.

	Younger Child		Older Child		Adult	
	Male	Female	Male	Female	Male	Female
n	15	15	15	15	12	12
Age range (years)	5–8	5–8	9–12	9–12	19–23	19–23
Age mean (years)	7	6	10	10		
Age SD (years)	1.8	1.4	1.2	1.0		

Adult prototypes were created in Psychomorph in the same way as the child face prototypes. 12 individuals’ faces were included in prototypes for each gender. Sad, angry, disgusted and fearful expressions were caricatured to create more intense expressions. For the happy and surprised expressions, caricaturing the prototype had not been necessary to make the expression high intensity. In order to standardise the number for images for each emotion used in this study, reduced intensity happy and surprised expressions were also included. These were created by decreasing the difference of the expression prototype from the ambiguous prototype to create a less intense expression.

**Fig 1:**
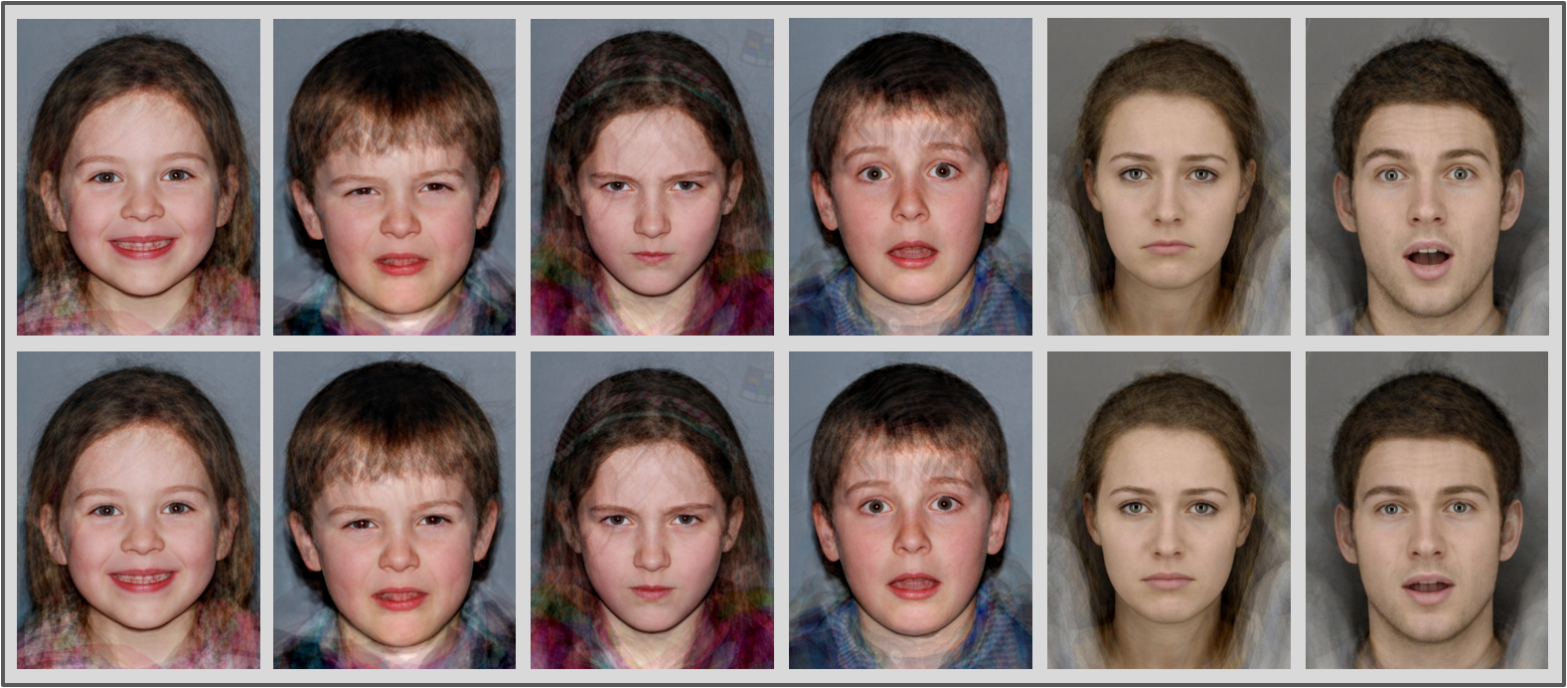
Examples of the 6 face prototypes each showing one of the 6 emotional expressions at each intensity. From left to right: age 5–8 female happy, age 5–8 male disgusted, age 9–12 female angry, age 9–12 male afraid, adult female sad, adult male surprised. The top row contains the the more intense versions and the bottom row contains the less intense versions. It is not possible to identify any individual's face from the final prototypes, therefore we did not require permission to publish these images.

There were therefore a total of 72 prototypical facial expression images: two intensity levels (high and low) of 6 emotions (happy, sad, angry, fearful, surprised and disgusted) on 6 face types (aged 5–8 female, aged 5–8 male, aged 9–12 female, aged 9–12 male, adult female and adult male). All images were cropped to 350 × 457 pixels. See Fig 1 for examples.

### Procedure

Testing was carried out on iPad tablet computers and took place at a table in the corner of the exhibition space at the science centre. Up to three participants were tested simultaneously. Recruitment and testing was carried out by 2 researchers. At the start of each testing session parents were asked to consent to their own/their child’s participation in the study and to report the ages and sexes of the children and/or parents taking part. Participants were instructed that their task was to select the emotion label that went with the face that they saw, and that there would be the choice of 6 emotions: happy, sad, angry, surprised, scared and disgusted. The label “scared” was used instead of “fear” because it was noted that children were more familiar with this word during the photograph collection phase. If children said that they did not understand what a word meant, clarification was given (e.g., “When something tastes or smells horrible you might feel disgusted”). Participants were told that the faces would appear quickly and that if they did not know the answer they should have a guess. Parents were instructed not to assist their children; however, in some cases parents did give help. The experimenter made a note of when this happened and data from those participants were excluded. Each of the 72 images was presented once and the order of presentation was randomised. In each trial, participants saw a fixation cross appear in the centre of the screen for a variable time between 1,500 and 2,500 ms, this variability was included to ensure participants did not get into a fixed response pattern. A face was then presented for 150 ms, followed by a mask of visual noise for 250 ms. The 6 emotion labels then appeared in a circle on the screen. The labels stayed on the screen until the participant had responded by touching one of the labels, at which point the next trial started. The position of the emotion labels was randomly selected for each participant and stayed the same throughout the testing session. There was a counter in the corner of the screen that gave the number of trials left out of 72.

### Data analysis

Participants were split into 3 age groups: 5–8 years (N = 33), 9–13 years (N = 70) and adults (N = 92) to match the ages of the face stimuli. There were 11 participants between the ages of 14–17 years who did not fit clearly in to any group. These individuals were included in the adult group, as after the age of 14 skeletal shape and size becomes more adult-like [21]. Analyses were repeated without these participants included, and there were no qualitative differences to the findings reported below. To test how expression recognition accuracy was affected by the age of composite face, the age of participant, the emotion, and the intensity of expression, a mixed 4-way ANOVA was performed. Face age with 3 levels, emotion with 6 levels, and intensity with 2 levels were within subjects factors, and participant age group with 3 levels was a between subjects factor. Participant sex was included as a covariate as this has been shown to affect emotion recognition in previous research [22].

Post-hoc tests were performed to determine the nature of interactions found in the main analysis. Separate 3 × 3 × 2 ANOVAs for each emotion, with face age, participant age group, and intensity as factors allowed us to determine for which emotions recognition was being affected by face age, age group and intensity. Separate 3 × 6 × 2 ANOVAs for each participant age group, with face age, emotion and intensity as factors allowed us to determine for which age group recognition was being effected by intensity.

A power calculation was completed after data collection in order to determine the size of interaction effect that could be detected with the sample size which we achieved. This calculation indicated that with our sample size (N = 195) we would be able to detect an interaction between face age and participant age group with an effect size of *F* = 0.127 with 95% power. This suggests that we had good power to detect even small interaction effects caused by an own-age advantage.

The data that form the basis of the results presented here are archived on the data.bris Research Data Repository (http://data.bris.ac.uk/data/), doi: 10.5523/bris.14hx5dbefrz1p1poxhfhgvltng.

## Results

ANOVA provided strong evidence for a main effect of participant age group (*F* [2, 191] = 25.485, *p* <. 001, ɳ^2^ = .211) and emotion (*F* [5, 955] = 22.657, *p* <. 001, ɳ^2^ = .106), but not face age (*F* [2, 382] = 2.265, *p* = .105, ɳ^2^ = .012). There was some evidence for a main effect of intensity (F [1, 191] = 3.634, *p* = .058, ɳ^2^ = .019). There was strong evidence for two-way interactions between participant age group and emotion (*F* [10, 955] = 3.060, *p* = .001, ɳ^2^ = .031), face age and emotion (*F* [10, 1910] = 6.596, *p* <. 001, ɳ^2^ = .033), intensity and age group (F [2, 191] = 3.679, *p* = .027, ɳ^2^ = .037), and intensity and emotion (F [5, 955] = 3.150, *p* = .008, ɳ^2^ = .016). However, there was no clear evidence for an interaction between face age and participant age group (*F* [4, 382] = 1.727, *p* = .143, ɳ^2^ = .018) or face age and intensity (F[2, 382] = 0.763, *p* = .467, ɳ^2^ = .004). There was also no evidence for any higher order interactions (*p*s >. 119)

**Fig 2:**
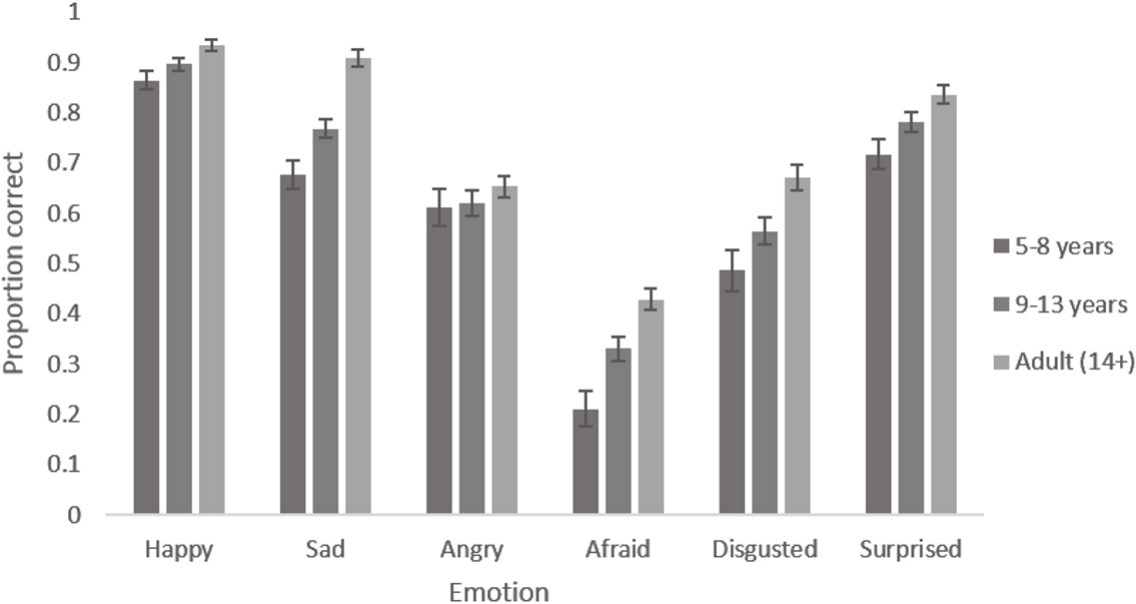
Mean proportion of correct responses to images showing each emotion by each participant age group. Error bars show standard error.

**Fig 3:**
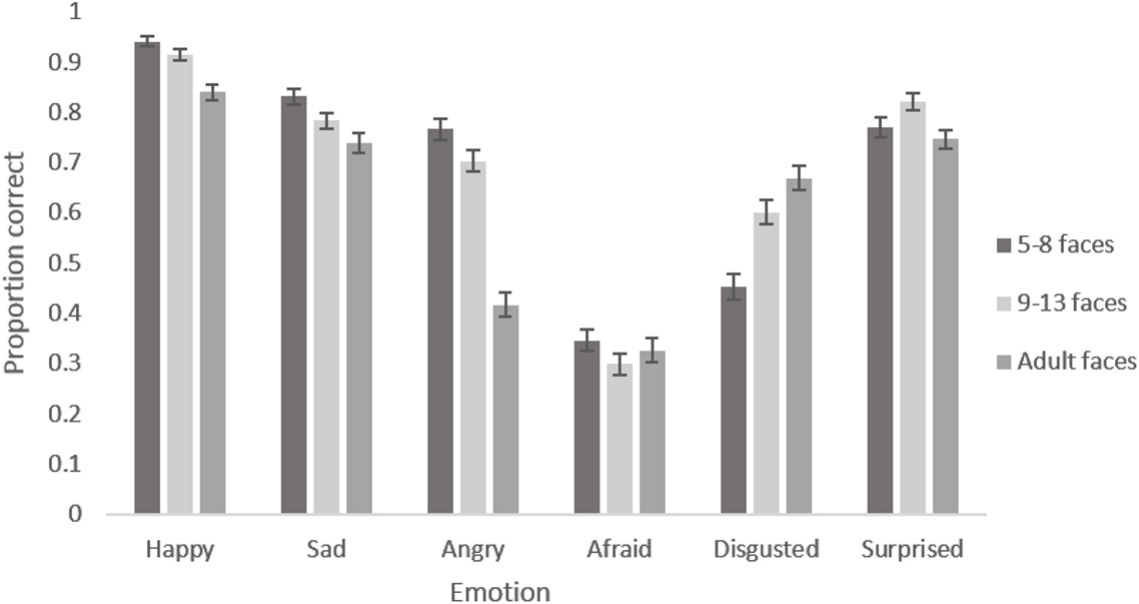
Mean proportion of correct responses to images showing each emotion from each age face set. Error bars show standard error.

**Fig 4:**
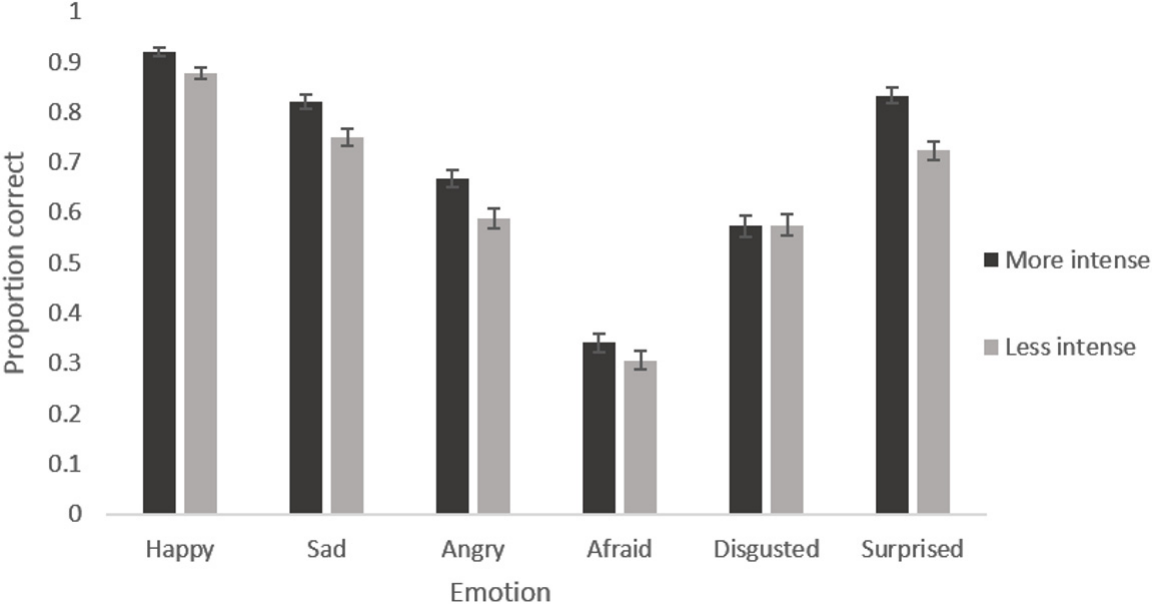
Mean proportion of correct responses to images showing each emotion at each intensity level. Error bars show standard error.

The main effect of participant age reflects the expected increase in accuracy of recognition with age. Adults were most accurate, followed by 9–13 year olds and then by 5–8 year olds for every emotion (see Fig 2). The main effect of emotion reflects the fact that certain emotions were recognised with greater accuracy than others. Happiness was recognised most accurately, followed by sadness and surprise, then anger and disgust, with fear being the least well recognised in every face age set (see Fig 3). The marginal main effect of intensity reflects the fact that accuracy was greater for the more intense expressions (see Fig 4). The lack of a main effect of face age shows there was no meaningful difference in overall accuracy of recognition of expressions on different aged faces.

**Fig 5:**
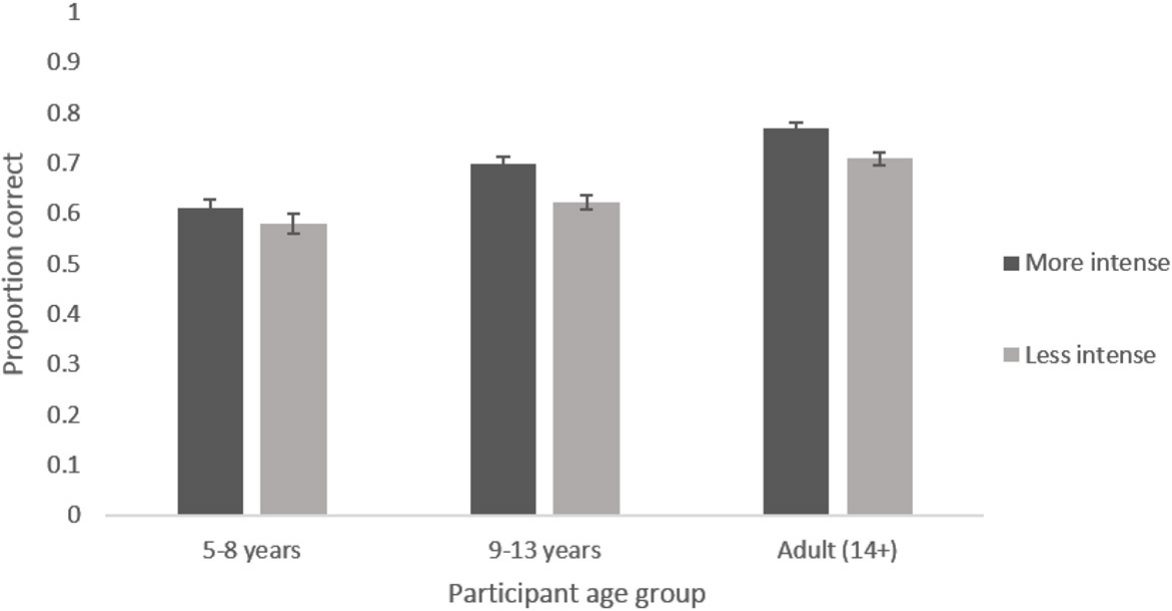
Mean proportion of correct responses to images at each intensity level by each participant age group. Error bars show standard error.

The interaction between participant age and emotion reflects the fact that improvement in recognition accuracy with age was greater for some emotions than others (see Fig 2). Post-hoc tests revealed clear evidence for age related improvements in accuracy for recognising all emotions (*F*s [2, 192] > 5.152, *p*s <. 007) except anger (*F* [2, 192] = 0.918, *p* = .401). The interaction between face age and emotion reflects the fact that happy, sad and angry expressions were recognised with greater accuracy on younger as opposed to older face prototypes while disgusted was recognised with greater accuracy on older as opposed to younger face prototypes. There was no clear pattern for fear and surprised (see Fig 3). Post-hoc tests revealed clear evidence for an effect of face age on the recognition of all emotions (*F*s [2, 384] > 5.932, *p*s <. 003) except fear (*F* [2, 384] = 1.418, *p* = .244). The interaction between intensity and emotion reflects the fact that there is a larger increase in recognition with increased intensity for some emotions compared to other emotions (see Fig 4). Post hoc tests reveal clear evidence for an effect of intensity on the recognition of all emotions (*F*s [1, 192] > 14.446, *p*s <. 001) except fear and disgust (*F*s [1,192] < 3.323, *p*s >. 070) The interaction between intensity and age group reflects the fact that there was a smaller increase in accuracy of recognition with increased expression intensity in the 5–8 year old group compared to the older age groups (See Fig 5). Post hoc tests confirmed that there was weaker evidence for an effect of intensity in the 5–8 year old group (*F* [1, 32] = 4.021, *p* = .053) compared to the 9–12 year old group (*F* [1, 69] = 63.621, *p* <. 001) and the adult group (*F* [1, 91] = 47.711, *p* <. 001).

**Fig 6:**
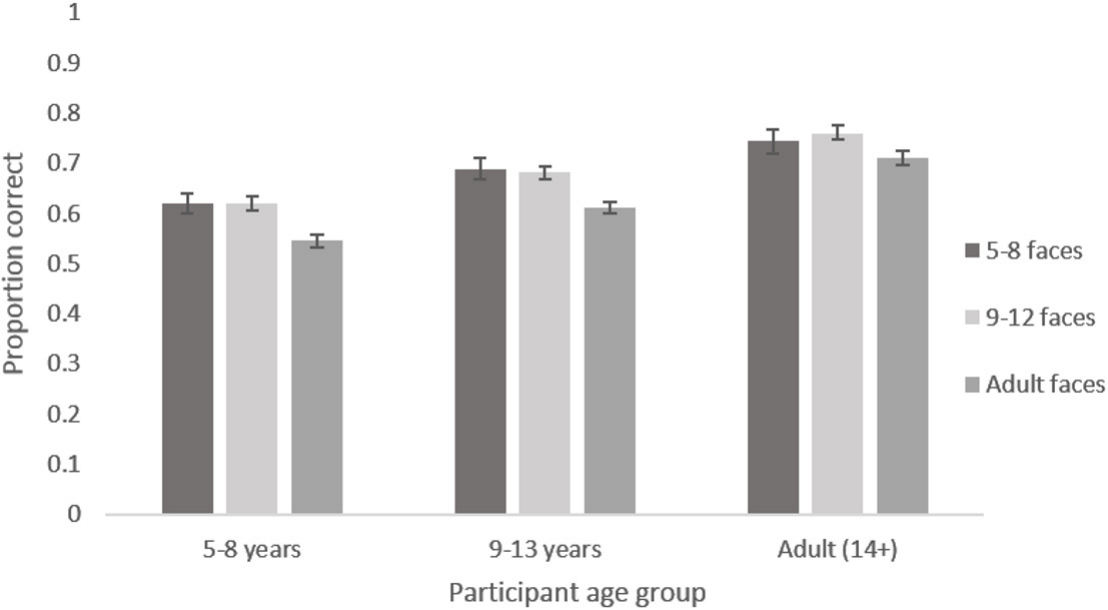
Mean proportion of correct responses to images from each age face set by each participant age group. Error bars show standard error.

The lack of interaction between face age and age group shows that recognition accuracy for expressions on the different age faces did not depend on participant age group. It was not the case that participants were more accurate at recognising emotions on faces that were drawn from their own age range (Fig 6).

## Discussion

In the current study we created prototypes of younger and older child faces displaying 6 emotional expressions. We then tested a large number of participants from a wide age range on their ability to recognise these and prototypical adult facial expressions in a forced choice emotion labelling paradigm. This allowed us to test whether the age of faces affected the ability of participants of different ages to recognise emotional expressions. We found that expressions were recognised as accurately on the children’s face prototypes as on the adults’ face prototypes, by both children and adults, suggesting there is no own-age advantage in children’s emotion recognition.

This is the first study to look at whether there is an own-age advantage in emotion recognition in children. We predicted that we would find evidence for an own-age advantage in emotion recognition, as there is evidence for an own-age advantage in children’s facial identity recognition [23] and evidence for other in-group advantages in facial emotion recognition [6]. There is also some evidence for an own-age advantage in older adults’ recognition of emotion expressions [10]. Our results are, however, consistent with two previous studies of own-age bias in emotion recognition that focused on older adults and found no evidence of an own-age bias in that age group [9, 11]. The suggestion by that failure to find evidence of an own age bias in these two studies was the due to lack of power [10] cannot easily be applied to our own study. We had a large sample which provided us with sufficient power to detect even small interaction effects, suggesting we were highly unlikely to miss evidence for an own-age advantage due to lack of power.

Certain characteristics of our adult age group limit the conclusions we can draw about own-age bias in adults’ emotion recognition. Firstly, adults in this study were predominantly parents of children within the age range of the child face prototypes. If in-group advantages in face processing exist because people become better at processing faces that they have had experience with [24, 25], we would not expect the adults in this study to perform less well at recognising emotion on children’s faces compared to adults’ faces, as they presumably have extensive experience recognising expression on their own children’s faces. Additionally, many of the adults’ ages did not match the age of the adult face prototypes, which were created from the faces of adult between 19 and 23 years of age, so adult participants may not have categorised our ‘adult’ face stimuli as belonging to their in-group. It is possible that if we had tested only young adults who lacked extensive experience of children’s faces, we may have seen an own-age advantage in the adult group. However, we also saw no own-age advantage for children recognising emotion in other children’s faces, despite the fact these children did not have an unusual amount of contact with young adults and the child stimuli were well matched in age to the ages of the child participants. We can therefore be more confident in concluding that there is no own-age advantage in children’s emotion recognition. Perhaps children do not develop an own-age in-group advantage because they have extensive experience of, and motivation for, processing adults’ faces. Children’s parents’ faces convey important affective information and children have a great deal of experience looking at their parents’ faces from a young age. It is therefore possible that an own-age advantage may exist for some age-groups which lack motivation for processing another age group’s faces (e.g., younger adults processing older adults’ faces) but not for children processing adults’ faces.

Considering the development of emotion recognition, our findings did replicate previous studies which have shown that emotion recognition is still developing in late childhood [1, 2]. Interestingly, we found improvements in accuracy of emotion recognition with age for all emotions except anger, suggesting a lack of development of anger recognition after early childhood. This has been shown in some other studies [14, 15] and may suggest that anger is a particularly easy emotion to learn.

The intensity of the expression prototypes was increased during stimuli development to ensure that the expressions were strong enough to be accurately recognised. Both original images and increased intensity images were presented in the study to look at whether this small manipulation was successful in increasing recognition accuracy. Our results show that higher intensity images were indeed recognised more accurately, although this difference was greater for some emotions than for others. This is unsurprising as the intensity of the expressions at each level were not standardised across stimuli. An unpredicted finding was that the younger child age group benefited less from increased intensity than the older child and adult age groups. This may be explained by increases in perceptual sensitivity to emotional expressions with age between 5 and 9 years of age [2]. Perhaps younger children were not sensitive to the subtle difference in intensity between the higher and lower intensity levels. The current study is somewhat limited in what it can tell us about sensitivity to expressions as only two levels of intensity were included. Further studies using more levels of intensity would be needed to fully understand how the intensity of expression affects recognition in different age groups.

A further potential limitation of the current study is that the stimulus set was not independently validated (e.g., by collecting ratings of strength and intensity of expression) before being used. However the results show that the adult participant group labelled the emotions on all three prototypes ages with an accuracy of between 71%-78%. This level of accuracy is equivalent to the overall mean accuracy found in validations of emotional expression stimulus sets in adult populations [26, 27], suggesting that our stimulus set was comparable to other stimulus sets. There was considerable variation in how well different emotions were recognised. However this is common in emotion stimulus sets and the pattern of differences evident in the current study, with”happy” being most accurate and “afraid” least accurate, is not unusual [27, 28]. These findings therefore suggest that our face prototype stimuli are valid depictions of emotional facial expressions.

There was also some variation in how well certain expressions were recognised on faces of different ages. Sadness, anger and happiness, were better recognised on younger face prototypes, whereas disgust was better recognised on older face prototypes. There was no clear pattern for surprise and fear. This may be due to differences in the intensity/clarity of expressions that the child models produced compared to the adult models. Anecdotally, we noticed that children were more likely to exaggerate expressions for the photographs, particularly the angry expression. Alternatively, it may be that certain expressions are more readily recognised on child faces as opposed to adult faces due to age related differences in the shape of facial features [8].

In conclusion, we found no evidence of an own-age bias in emotion recognition in child and adult participants using younger child, older child, and young adult emotional expression prototypes. Instead we found that emotions were recognised as accurately on child age and adult age faces by all age groups. These results strongly suggest a lack of own-age bias in emotion recognition in children. However further studies with greater numbers of adult subgroups and a better match between the ages of adult participants and the ages of adult face stimuli, will be needed to confirm a lack of own-age bias in emotion recognition in adults.
