# Correction: No Own-Age Advantage in Children's Recognition of Emotion on Prototypical Faces of Different Ages

**DOI:** 10.1371/journal.pone.0131488

**Published:** 2015-06-22

**Authors:** Sarah Griffiths, Ian S. Penton-Voak, Chris Jarrold, Marcus R. Munafò

The following information is missing from the Funding section: Marcus Munafò is supported by the MRC Integrative Epidemiology Unit at the University of Bristol.
